# Autologous bone marrow derived mesenchymal stem cells are safe for the treatment of Achilles tendinopathy

**DOI:** 10.1038/s41598-024-61399-3

**Published:** 2024-05-19

**Authors:** Andrew J. Goldberg, Lorenzo Masci, Paul O’Donnell, Ruth Green, Deirdre Brooking, Paul Bassett, Mark W. Lowdell, Roger K. W. Smith

**Affiliations:** 1 Division of Surgery, UCL Institute of Orthopaedics & Musculoskeletal Science, Royal Free Hospital, 9th Floor (East), 2QG, 10 Pond St, London, NW3 2PS UK; 2 Department of Research and Innovation, Royal National Orthopaedic Hospital (RNOH), Brockley Hill, Stanmore, Middlesex UK; 3 MSK Lab, Faculty of Medicine, Department of Surgery & Cancer, Imperial College London, Level 2, Faculty Building, South Kensington Campus, London, SW7 2AZ UK; 4 The London Ankle & Arthritis Centre, The Wellington Hospital, Wellington Place, London, NW8 9LE UK; 5 Institute of Sport Exercise and Health, Tottenham Court Road, London, UK; 6 Department of Radiology, Royal National Orthopaedic Hospital NHS Trust, Brockley Hill, Stanmore, HA7 4LP UK; 7 Statsconsultancy Ltd., 40 Longwood Lane, Amersham, Bucks, HP7 9EN UK; 8 Centre for Cell, Gene & Tissue Therapeutics, Royal Free Hospital, Pond Street, London, NW3 2QG UK; 9 Department of Veterinary Clinical Sciences and Services, The Royal Veterinary College, Hawkshead Lane, Hatfield, Hertfordshire, AL9 7TA UK

**Keywords:** Stem cells, Health care

## Abstract

Achilles tendinopathy is a disabling condition that affects more than 50% of runners. Pre-clinical studies in a large animal model of naturally-occurring tendinopathy similar to human Achilles tendinopathy has shown benefits of autologous bone marrow-derived mesenchymal stem cell (MSC) implantation. However, MSCs are advanced therapies medicinal products (ATMPs), with strict regulatory requirements. Guided by the regulator we carried out a first in man study to assess the safety and efficacy of autologous MSC injection in human patients with non-insertional Achilles tendinopathy. Ten patients, mean age 47 with mid-portion Achilles tendon pain and swelling for more than 6 months, underwent autologous cultured cell injections (median 12.2 × 10^6^, range 5–19 × 10^6^ cells) into their Achilles tendon. At 24 weeks follow-up, no serious adverse reactions or important medical events were observed. MOXFQ, EQ-5D-5L, and VISA-A scores improved clinically at 12 and 24 weeks. VAS pain improved increasingly at 6, 12 and 24 weeks. MOXFQ Pain and VISA-A Scores improved > 12 points from baseline to 24 weeks in 8 patients. Maximum anteroposterior tendon thickness as measured by greyscale US decreased by mean 0.8 mm at 24 weeks. This phase IIa study demonstrated the safety of autologous MSC injection for non-insertional Achilles tendinopathy and provides proof-of-concept of the technique in patients, all of whom had previously failed conservative treatments for chronic disease and leads the way for a larger randomised controlled trial.

## Introduction

Achilles tendinopathy (AT) is a degenerative process resulting in chronic pain and dysfunction. AT has a major prevalence in the general and sporting community^1,2^ The management of Achilles tendinopathy is a challenge. The main non-operative management is with exercises, orthotics, acupuncture, physiotherapy^3–5^ and shockwave therapy^6^. Most patients receive multiple treatments over time and up to 45% of patients consider surgery at some stage^4^. Good data on safe and effective non-surgical treatments are lacking.

The underlying mechanism of pain in AT is controversial. The contribution of mechanical, biochemical, and inflammatory factors is uncertain. Evidence of neovascularisation (ingrowth of blood vessels and accompanying nerves) in the area of tendon changes has been observed^2,25^. However, the role of neovascularisation as a consequence or cause of pain remains uncertain. High concentrations of lactate^1^ and the neurotransmitter glutamate^4^ have been reported in chronic painful AT.

Mesenchymal stem cells (MSCs) are stromal cells with ability to self-renew and differentiate into multiple lineages, as well as act via the release of paracrine factors to modulate inflammation^7^. They can be isolated from a variety of tissues, such as umbilical cord, bone marrow, and adipose tissue^7^. MSCs have shown promise in heart disease^8^, musculoskeletal disorders^9^, and neurological disorders^10^.

Pre-clinical studies in a large animal model of naturally-occurring tendinopathy similar to human Achilles tendinopathy^11^ have shown significant benefits of autologous bone marrow-derived MSC implantation^12,13^.

In human tendon studies, various MSCs have been used for patella tendinopathy^14^; Achilles tendinopathy^15,16^; and tendon ruptures of the Achilles^17^ and shoulder rotator cuff^18,19^. However, to our knowledge, there have been no trials conducted using autologous cultured bone marrow derived MSC’s (BMMSC) for the treatment of Achilles tendinopathy.

Any human treatment involving cell-based products is considered an advanced therapies medicinal product (ATMP), with strict regulatory requirements^20^. Regulators such as the Food & Drug Administration (FDA), European Medicines Agency (EMA), and UK Medicines and Healthcare Products Regulatory Agency (MHRA) require a stepwise approach to the responsible introduction of new cell-based treatments following preclinical bench-top testing. In discussion with the MHRA, we conducted a first-in-human study to determine the safety and efficacy of autologous cultured BMMSC injection for AT. The regulator recommended including between 8 and 12 patients, to assess a primary outcome of serious adverse reactions, with secondary outcomes of clinical measure scores and ultrasound measurements. The aim was to produce the requisite early-stage safety data to inform a larger randomised controlled study.

## Results

### Patients

**Figure 1:**
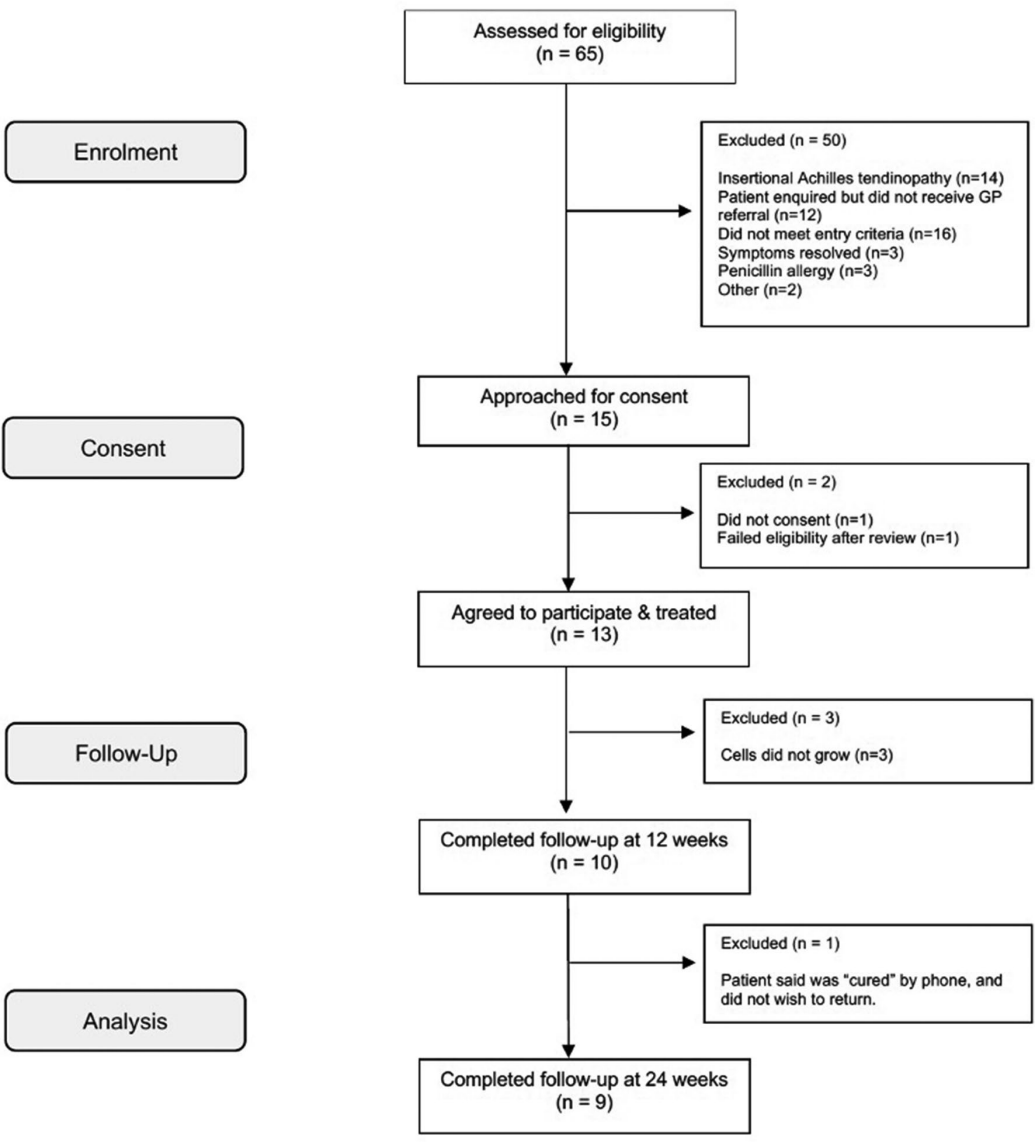
CONSORT Diagram.

Of 65 patients with AT > 6 months duration and assessed for eligibility, 50 patients were excluded (Fig. 1). The remaining 15 patients were approached for consent. One declined to participate, one failed to meet eligibility requirements after a more detailed review, and three underwent MSC harvest, but their cells did not grow sufficiently to proceed with implantation. Thus 10 patients (median age 47, range 37–53 years; 5 (50%) males and 5 females) underwent purified autologous BMMSC injection (median 12.2 × 10^6^, range 5–19 × 10^6^ cells) and completed the 24-week follow-up.

### Primary outcome: safety

No serious adverse reactions or important medical events were observed (incidence 0%, 95% confidence interval, CI 0% to 31%). No complications due to MSC harvest or injection procedures were observed.

### Clinical outcome measures

Patient-reported clinical outcome measures at baseline and 6, 12 and 24 weeks post-injection are summarized in Table 1. All three MOXFQ subscales, where a lower score represents a better outcome, improved significantly at 6, 12 and 24 weeks versus baseline, aside from the social interaction subscale from baseline to 6 weeks. The MOXFQ pain score improved from mean (± sd) 52 ± 11 at baseline to 24 ± 18 by 24 weeks (p < 0.001). The change in MOXFQ pain score exceeded the minimal clinically important difference (MCID) of 12 points in 8 of 10 patients.

**Table 1:** Clinical Outcome Measure Scores shown as mean ± standard deviation, and change from baseline shown as mean with 95% confidence interval in brackets.

Outcome measure	Baseline	6 weeks			12 weeks			24 weeks			p-value (baseline to 24 weeks)
	Mean ± SD	N	Mean ± SD	Change^^^	N	Mean ± SD	Change^^^	N	Mean ± SD	Change^^^	
MOXFQ walking	53 ± 23	10	40 ± 21	− 13 (− 24, − 2)	10	23 ± 17	− 30 (− 43, − 17)	9	25 ± 18	− 27 (− 41, − 13)	0.002
MOXFQ pain	52 ± 11	10	40 ± 15	− 12 (− 18, − 5)	10	29 ± 15	− 23 (− 32, − 14)	9	25 ± 18	− 28 (− 41, − 15)	0.001
MOXFQ social	43 ± 18	10	36 ± 19	− 7 (− 14, 0)	10	26 ± 17	− 18 (− 29, − 6)	9	24 ± 17	− 17 (− 28, − 6)	0.006
EQ-5D-5L*	0.70 [0.70, 0.73]	9	0.74 [0.69, 0.79]	0.02 [0.00, 0.18]	9	0.77 [0.69, 0.82]	0.07 [0.00, 0.27]	8	0.77 [0.70, 0.96]	0.16 [0.00, 0.46]	0.03
EQ-VAS overall health	71 ± 14	9	77 ± 9	7 (0, 14)	9	80 ± 11	10 (2, 18)	7	82 ± 11	14 (8, 20)	0.001
VISA-A	41 ± 22	10	47 ± 21	7 (1, 13)	10	59 ± 16	19 (12, 26)	9	61 ± 21	24 (13, 34)	< 0.001
VAS pain	43 ± 23	10	36 ± 21	− 7 (− 20, 6)	10	26 ± 24	− 17 (− 32, − 2)	9	22 ± 19	− 24 (− 44, − 3)	0.03
SAS	5.0 ± 2.4		n/a	n/a		n/a	n/a	8	6.6 ± 1.8	1.6 (0.4, 2.9)	0.02

*n/a* not applicable.

^^^Reported as mean change with 95% confidence intervals.

*Reported as median [inter-quartile range] at each time point, with median change [95% CI].

EQ-5D-5L and EQ-VAS scores improved significantly from baseline to each subsequent timepoint. At 24 weeks, EQ-5D-5L scores increased by median 0.16 units versus baseline, while EQ-VAS scores increased by mean 14 units. Similar improvements were observed in VISA-A scores, which increased from baseline with greater differences at each subsequent time point, and mean increase of 22 units at 24 weeks (p < 0.001). VAS pain scores were significantly lower at 12 and 24 weeks versus baseline, with mean reduction of 23 units (p = 0.03) at 24 weeks. The SAS increased from baseline to 24 weeks by mean 1.6 units (p = 0.02). One patient refused to attend the final review as he informed the trial manager, he felt he was “cured” and back to running. Although all the other 9 patients completed questionnaires at 24 weeks, not all questions were answered.

### Successful outcomes

At 24 weeks, 8 of 10 patients (80%; 95% CI 44% to 97%) had a successful outcome, defined as changes in outcome scores from baseline meeting or surpassing the MCID (i.e., > 12 points on the MOXFQ Pain Subscale and VISA-A).

### Ultrasound outcomes

Ultrasound outcomes were determined separately by each radiologist. The maximum AP thickness of the tendon did not vary significantly from baseline through 12 weeks (Table 2), but decreased by a mean of 0.8 mm at 24 weeks (p = 0.02). Distance of the lesion from the calcaneum dorsal cortex, size of focal change, and percentage of disorganised tissue (i.e., type of fibre) did not vary significantly from baseline through 24 weeks. Furthermore, there was no change in the Doppler assessment of neovascularization over 24 weeks. UTC was only obtained on 6 of the 10 patients at 24 weeks due to logistical challenges but no significant changes in fibre type was observed (Fig. 4).

**Table 2:** Ultrasound outcomes, shown individually for each radiologist (Rad), and UTC outcomes, reported as mean ± standard deviation, unless noted otherwise.

Outcome	Rad	Baseline	6 weeks			12 weeks			24 weeks			P-value (baseline to 24 weeks)
			N		Change mean (95% CI)	N		Change mean (95% CI)	N		Change mean (95% CI)	
Max AP Thickness, mm	1	8.2 ± 1.5	5	8.5 ± 1.3	0.4 (− 0.1, 0.8)	4	8.0 ± 1.6	0.0 (− 0.1, 0.1)	2	–	–	
	2	9.2 ± 1.6	8	9.2 ± 1.7	0.0 (− 0.3, 0.4)	7	8.6 ± 2.1	− 0.1 (− 0.5, 0.3)	5	7.8 ± 1.3	− 0.8 (− 1.3, − 0.2)	0.02
Lesion distance, mm	1	39 ± 8	5	40 ± 7	2 (− 2, 5)	4	36 ± 7	− 2 (− 6, 3)	2	–	–	
	2	40 ± 17	8	45 ± 9	5 (− 8, 18)	7	40 ± 10	0 (− 4, 4)	5	43 ± 6	10 (− 15, 34)	0.34
Focal change,* mm	1	0.0 [0.0, 3.6]	5	0.3 [0.0, 3.6]	0.0 [− 3.3, 3.0]	4	1.5 [0.0, 3.7]	0.1 [0.0, 3.0]	2	–	–	
	2	0.0 [0.0, 5.3]	8	0.3 [0.0, 3.0]	0.0 [− 3.2, 0.7]	7	0.3 [0.0, 2.3]	0.0 [− 4.6, 0.7]	5	0.3[0.0, 3.0]	0.0 [0.0, 0.0]	1.00
% thickness*	1	0 [0.42]	5	3 [0, 39]	0 [− 39, 38]	4	21 [0, 44]	1 [0, 42]	2	–	–	
	2	0 [0, 54]	8	0 [0, 35]	0 [− 29, 7]	7	0 [0, 38]	0 [− 45, 10]	5	0 [0, 0]	0 [0, 0]	1.00
UTC fibre type^^^ (% disorganized)	–	32 ± 16	7	29 ± 9	− 3 (− 17, 11)	7	26 ± 7	− 2 (− 17, 14)	6	27 ± 14	− 3 (− 25, 19)	0.73

*Rad* radiologist, *Max AP Thickness* maximum anteroposterior thickness, *UTC* Ultrasound tissue characterization.

*Reported as median [inter-quartile range] at each time point, along with median change [95% CI].

^^^Disorganized = Type 3 (i.e. haphazard fibres aligned) + Type 4 (i.e. amorphic material or no fibres.

Inter-observer agreement for conventional ultrasound measurements was good for calculation of maximum AP thickness (ICC 0.95, 95% CI 0.87, 0.98), lesion distance from the calcaneum (ICC 0.89, 95% CI 0.76, 0.95) and level of neovascularisation (kappa 0.82, 95% CI 0.50, 1.00), but was poor for determining size of focal change (ICC 0.41, 95% CI 0.00, 0.71) and percentage maximum tendon thickness (ICC 0.38, 95% CI 0.00, 0.69).

## Discussion

This phase IIa, proof-of-concept, single-arm study determined the safety of autologous BMMSC injection for AT in a clinical setting. No serious adverse reactions or important medical events were observed in all 10 patients. In these patients, who had previously failed conservative treatments for chronic AT, treatment with autologous BMMSC injection provided relief of symptoms and notable improvement in MOXFQ scores, EQ-5D-5L and EQ-VAS scores, and walking improved through 24 weeks post-injection.

Existing treatments relying on inflammation-repair sequences have shown equivocal results^5,21,22^ and so a regenerative solution is conceptually attractive. The mechanism of action of MSCs is not fully understood. It may be tropic, such that MSCs secrete paracrine factors to coordinate the body’s response, including an immunomodulatory role^23^, or it may be the direct action of stem cells becoming tendon cells^13^. We do not know if the observed benefit is due to direct effects, interactions relating to the MSCs, or a placebo effect, as this study was not controlled.

This study used autologous cells. Ilic and Atkinson^15^ investigated the injection of allogenic MSCs derived from human placenta into the Achilles tendon in six patients was safe, with no adverse reactions four weeks following injection, but they did not evaluate clinical outcomes.

Ultrasound tissue characterization (UTC) is a reliable imaging method for clinical assessment of AT^24^. The dynamics of grey levels, using histomorphology of the tissue specimen as a reference, is strongly related to tendon matrix architecture and integrity^25,26^. While we observed a clinical benefit of BMMSC injection, imaging did not reveal conclusive changes in the structure of tendons injected with BMMSCs. The maximal anteroposterior thickness was significantly reduced by 0.8mm at 24 weeks (p = 0.02), but other ultrasound parameters and neovascularisation did not vary from baseline. Moreover, UTC showed no significant change in tendon matrix in the region injected with BMMSCs. As UTC is more reliable and objective than two-dimensional ultrasound at defining fiber alignment^25–28^, the clinical benefits observed here may not be secondary to improvements in tendon structure. Other possibilities for improved clinical outcome include changes in inflammation (and hence pain) or placebo-related changes. A larger study using a placebo control is required to determine whether the positive clinical effect is due to MSC injection or secondary to other effects such as needling or placebo.

In some groups there is a move towards one-step techniques such as bone marrow buffy coats (i.e., mononuclear cells)^16,29,30^, or adipose tissue derived cells^9^, which remove the need for culture expansion. It is important to appreciate that these techniques produce mixed cell populations and not isolated MSCs. Autologous bone marrow-derived concentrate (BMC) has also been used in tendon disease. Thueakthong et al. reviewed 10 patients with non-insertional Achilles tendinopathy treated with iliac crest derived cells and showed a trend to improvement over 48 weeks in an uncontrolled study^16^. BMC have also been used in Achilles tendon repairs and rotator cuff injuries, suggesting increased healing and better clinical scores^17–19^. However, their use remains controversial as allogenic BMC did not show significant improvements in clinical outcome in a model of rotator cuff repair^31^. The current study evaluated autologous, culture-expanded BMMSCs and not BMC and does not support nor detract from this work. Five to 19 × 10^6^ cells were used in this study, whereas the number of stem cells in techniques using non-expanded mononuclear cells may only be in the tens or hundreds. We appreciate that the two-stage process used here may be unattractive commercially; nevertheless it is important to note that allogenic stem cell banks have inherent risks of disease transmission and alloreactivity^32^. This study has demonstrated a milestone in safety for a two-stage treatment.

Limitations of this study include the lack of a control group and a small sample size, but this was deliberate in discussion with the regulator as a first-in-human study on an advanced therapies medicinal product. The MHRA deemed the sample size sufficient for demonstrating safety of the procedure in humans, and if at least 6 of 10 patients had positive clinical outcomes they felt this would inform a larger RCT to confirm efficacy. This study was, therefore, not powered to demonstrate statistically significant changes in clinical outcomes scores. Other limitations include the technical aspects of BMMSC culture including the potential confounding factor of the carrier media and our ability to comment on the ideal dosage dosage of BMMSCs. Based on our preclinical work^13^ we estimated that a minimum required dosage of 4 × 10^6^ were needed, although we also showed that the number of cells decreased with time from release to injection, and hence we agreed to begin with a much larger dose of 20 million cells anticipating cell loss. To deliver a pragmatic and clinically relevant trial patients in this study received a variable number of BMMSC’s ranging from 5 × 10^6^ to 19 × 10^6^ cells, with a median of 12.2 × 10^6^ cells. We did not observe any correlation between clinical outcome and number of cells injected.

In addition, it is possible that the physiotherapy administered could be a potential confounder. All patients in this study had completed a full physiotherapy programme before the intervention and then returned to the same physiotherapist for a further programme after the intervention and hence we believe the effects of differences in physiotherapy protocols to be negated.

In conclusion, this proof-of-concept study demonstrated the safety of autologous BMMSC injection for AT. The observed clinical improvements are encouraging and support a larger, phase IIb RCT to establish efficacy of this procedure.

## Methods

### Study design and patients

This prospective, phase IIa, proof-of-concept, single arm, open-label study was conducted at a tertiary referral hospital in the United Kingdom. Patients aged 18–70 years and referred to the hospital with pain and tender swelling in the mid-portion of the Achilles tendon (i.e., > 1 cm proximal to the insertion) for > 6 months and who had failed conservative treatment (including a full course of physiotherapy) were interviewed by the Chief Investigator and invited to participate in the study (inclusion and exclusion criteria in Table 3). This study was approved by the National Research Ethics Service Committee (London, Harrow; reference 13/LO/1670) and was registered at clinicaltrials.gov (NCT02064062) on 17/02/2014. Patients provided written, informed consent and all experiments were performed in accordance with relevant guidelines and regulations.

**Table 3:** Patient inclusion and exclusion criteria for study.

Inclusion criteria	Exclusion criteria
Age 18 to 70 years	Previous bony surgery at or in proximity to bone harvest site, or on Achilles tendon
Pain and tender swelling in mid-portion of Achilles tendon for > 6 months	Pregnant or lactating
Failed conservative treatment	Inflammatory arthritis
Eligible for surgery	Positive for hepatitis B, hepatitis C, HIV-1, HIV-2, syphilis, or human T-cell lymphotropic virus
Able to provide written, informed consent	Using steroids, antitumor necrosis factor drugs, methotrexate, or fluoroquinolones currently or within 3 months of assessment
	Known or suspected underlying haematological malignancy
	Another active malignancy in past 3 years
	Bovine or antibiotic allergy

Screening assessments included a pre-screening interview, medical history, and clinical examination of the foot and ankle for: limb alignment and biomechanical abnormalities; size and location of Achilles tendon swelling; range of motion of the ankles, hindfeet, midfeet and forefeet; gastrocnemius tightness; and to rule out other conditions. Tendon continuity was confirmed with the Simmonds-Thompson test^33^. Blood tests for viral infections (Table 3) were conducted 7–30 days prior to bone marrow harvest. Pregnancy tests were conducted within 7 days prior to bone marrow harvest.

### Imaging

**Figure 2:**
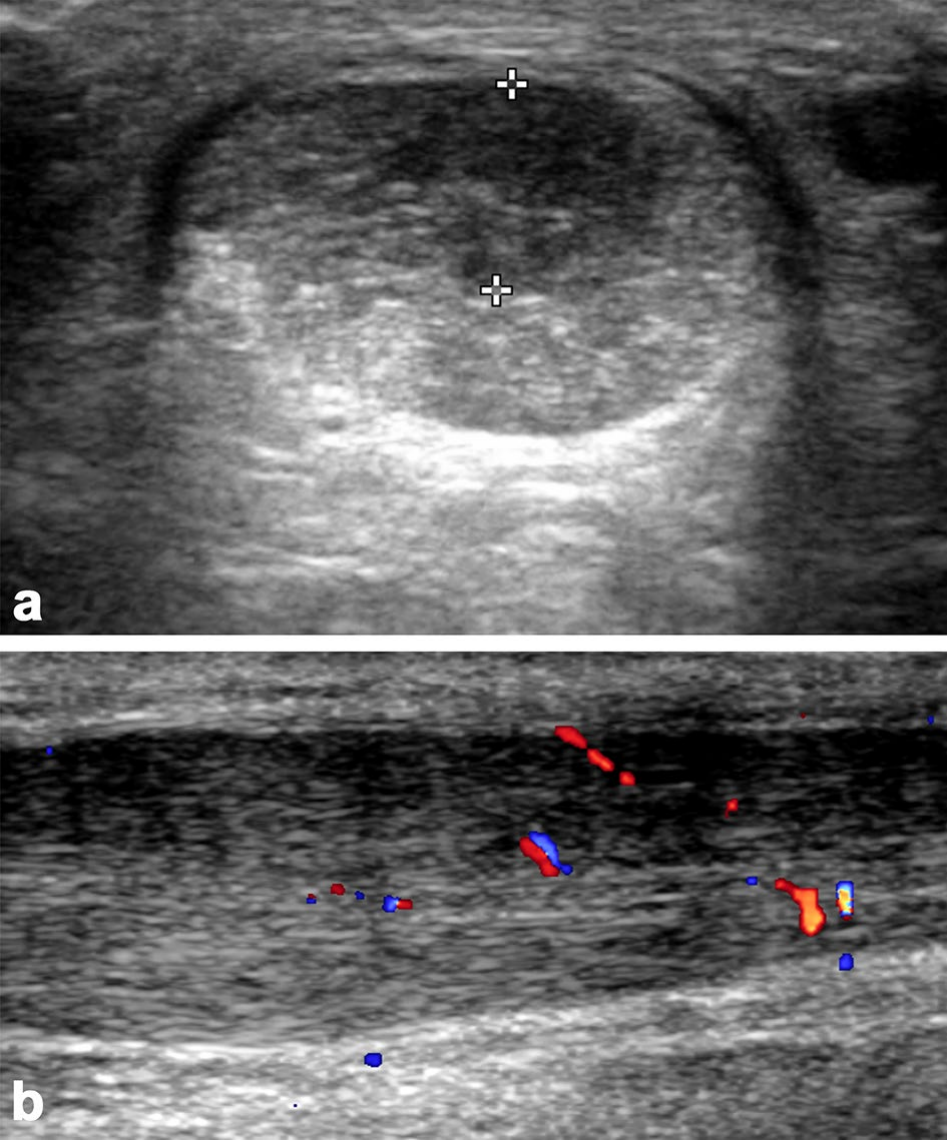
(**a**) Transverse greyscale ultrasound image of an enlarged Achilles tendon, with low reflectivity on its superficial aspect affecting approximately 50% of the anteroposterior diameter of the tendon. (**b**) Longitudinal ultrasound image with colour Doppler. The tendon is fusiform in morphology, consistent with chronic non-insertional tendinosis, and the superficial aspect of the tendon shows low reflectivity. Moderate (grade 3) vascularity is seen in the tendon.

Patients underwent routine conventional 2D greyscale ultrasound and ultrasound tissue characterization (UTC) prior to bone marrow harvest. Conventional ultrasound examinations were performed with the patient in prone position, feet hanging freely over the examination table edge. The foot was dorsiflexed by the examiner, aiming to reach approximately perpendicular to the tibia to reduce tendon redundancy. High resolution greyscale and colour Doppler ultrasound was performed using a GE Logiq E9 US unit (GE Medical Systems Ltd, Bucks, UK) with a ML 6-19MHz multifrequency linear array transducer. Ultrasound studies were independently performed by two experienced consultant musculoskeletal radiologists, consecutively within a few minutes of each other, the second radiologist blinded to the findings of the first. Measurements included: maximum anteroposterior (AP) tendon thickness (Fig. 2a); distance of maximum tendon thickening from the calcaneum dorsal surface; and size of focal tendinosis/tendon low reflectivity (i.e., AP measurement in mm, expressed as percentage of AP tendon thickness). The presence and degree of increased colour Doppler signal and/or neovascularity (Fig. 2b) was graded as (1) absent (no vessels); (2) mild (minimal vessels, usually at the tendon’s anterior aspect); (3) moderate (low level of vessels throughout the tendon); (4) marked (marked increased vasculature throughout).

The Achilles tendon was visualised tomographically with ultrasound tissue characterization (UTC; UTC Imaging, Stein, Netherlands)^28^. A high-resolution linear ultrasound probe (7–12 MHz, Teratech, USA) fixed to a tracking device moved along the tendon to collect adjoining transverse ultrasound images every 0.2mm over 12cm. The patient was positioned prone on the examination table with the ankle caudal to the edge. To achieve maximum passive dorsiflexion, the examiner pushed their knee against the patient’s forefoot. All scans were performed by one experienced examiner (LM), distally to proximally^34^. Images were captured, stored, and compounded to create a three-dimensional (3D) volume block for tomographical reconstructions (i.e., transverse, sagittal, coronal, and 3D coronal planes). Validated UTC algorithms (UTC 2010, UTC Imaging) discriminated four distinct echotypes of matrix integrity: Type 1: aligned fibrillar structure; Type 2: wavy fibres; Type 3: haphazard aligned fibres; and Type 4: amorphous material with no fibres^34^. For analysis, Types 1 and 2 represented organized tissue, and Types 3 and 4 represented disorganized tissue^28^. The percentage of each echotype was measured within the BMMSC injection region.

### Treatment procedure

The patient was placed in a left lateral decubitus position. Approximately 8mL of bone marrow aspirate was collected from the right posterior superior iliac crest into a heparinised syringe under local anesthetic and sent to a GMP facility licensed by the UK MHRA for production of human stem cells as investigational medicines. The patient was monitored after the procedure for adverse events and prescribed pain medication as indicated. Any adverse events during or immediately following bone marrow harvest were recorded.

Bone marrow-derived mesenchymal stem cells (BMMSCs) were isolated from bone marrow mononuclear cells by plastic adherence for 16–24 h using a standardized, GMP-compliant protocol of Dulbecco’s Modified Eagle Medium supplemented with 10% fetal bovine serum. The adherent cells were expanded through up to three passages over 28–43 days to obtain 4–20 million BMMSCs. A dose of 4 × 10^6^ cells was considered the minimum required for efficacy^12,13^, and the ideal dose achieved was 20 × 10^6^ cells. The actual dose varied between these values (median 12.2 × 10^6^, range 5 to 19 × 10^6^), dependant on culture conditions. The final, aseptic products were > 90% MSC (CD73 + /CD90 + /CD105 +), > 95% viable, and formulated as investigational medicinal products in suspension in two syringes ready for direct injection. The syringes were shipped by courier to the clinical site on the day of treatment.

**Figure 3:**
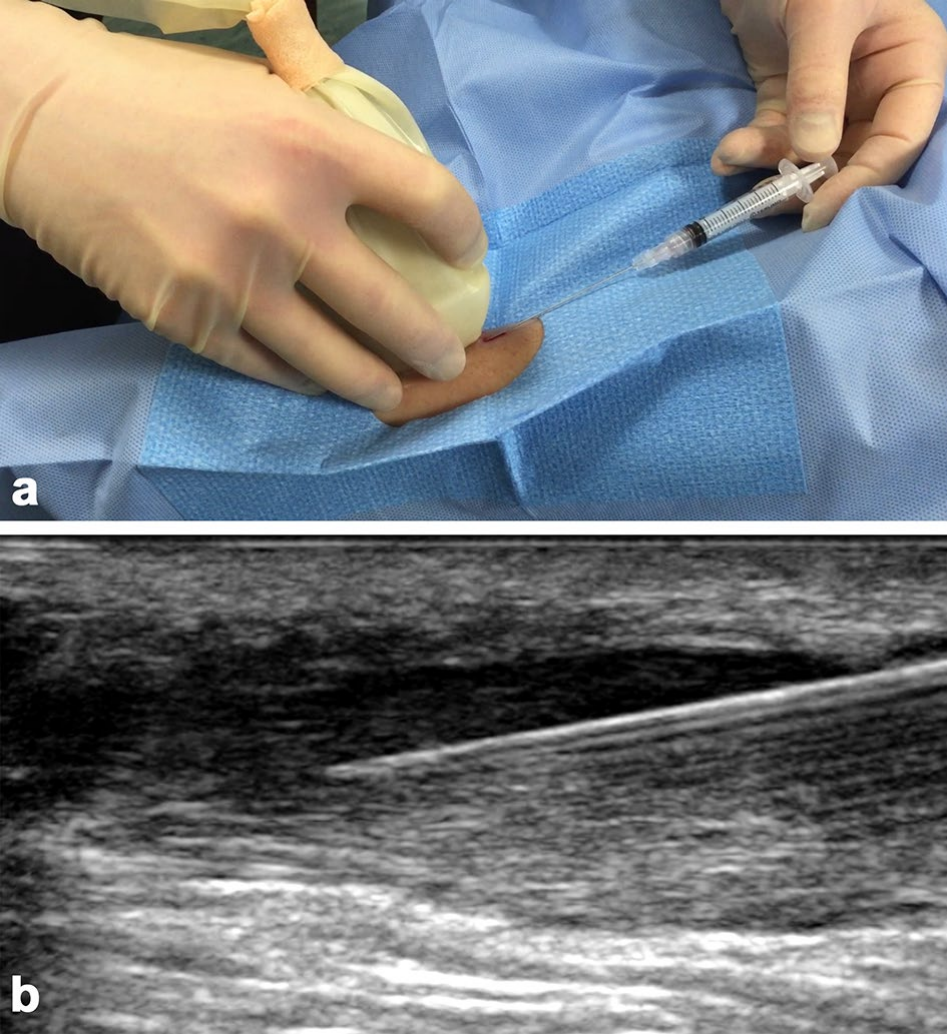
Stem cell injection performed under local anesthetic and greyscale ultrasound control: (**a**) intraoperative image; (**b**) longitudinal ultrasound image. The needle was inserted from distal to proximal and lies within the superficial low reflectivity region of the thickened tendon. Injection was performed while withdrawing the needle.

Stem cell injection was performed approximately 5 (range: 4–6) weeks after bone marrow harvest in an outpatient setting. A suspension of purified BMMSCs in 1 mL of Dulbecco’s Modified Eagle Medium was injected percutaneously under local anaesthetic using in-plane ultrasound guidance by an experienced, trained radiologist longitudinally along the length of the tendon. The area of maximal anteroposterior tendon thickness was identified, and the needle inserted 1 cm proximal to the lesion and passed across the abnormal tendon, ending 1 cm distal (Fig. 3). The injection was administered as the radiologist pulled the needle backwards through the abnormal part of the tendon. An adhesive, sterile, dry dressing was applied. Patients were monitored for at least 2 h following implantation for immediate adverse effects. Two days after the procedure, the trial coordinator telephoned all patients to check their well-being and record any adverse events.

Patients underwent a standard course of physiotherapy for Achilles tendinopathy, comprised of a 12-week eccentric loading programme^35^.

### Outcome assessments

Patient demographics, comorbidities and concomitant medications were recorded at baseline. Patients completed several outcome measures at baseline that represented the best methods we could identify to assess foot and ankle pathology and Achilles tendinopathy^36^: Victorian Institute of Sports Assessment Achilles (VISA-A) Questionnaire^37^, Visual Analogue Score (VAS) for pain (0–100 scale)^38^, Manchester Oxford Foot and Ankle Questionnaire (MOXFQ)^39,40^, EuroQol 5-dimensions 5-levels (EQ-5D-5L)^41^, and Stanmore Sporting Activity Scale (SAS) modified from the UCLA sports activity score^42^.

At 6, 12 and 24 weeks following MSC implantation, patients underwent clinical assessment, adverse event review, conventional ultrasound imaging, UTC, and completed the VISA-A, VAS pain, MOXFQ, and EQ-5D-5L. The SAS was completed at 24 weeks.

**Figure 4:**
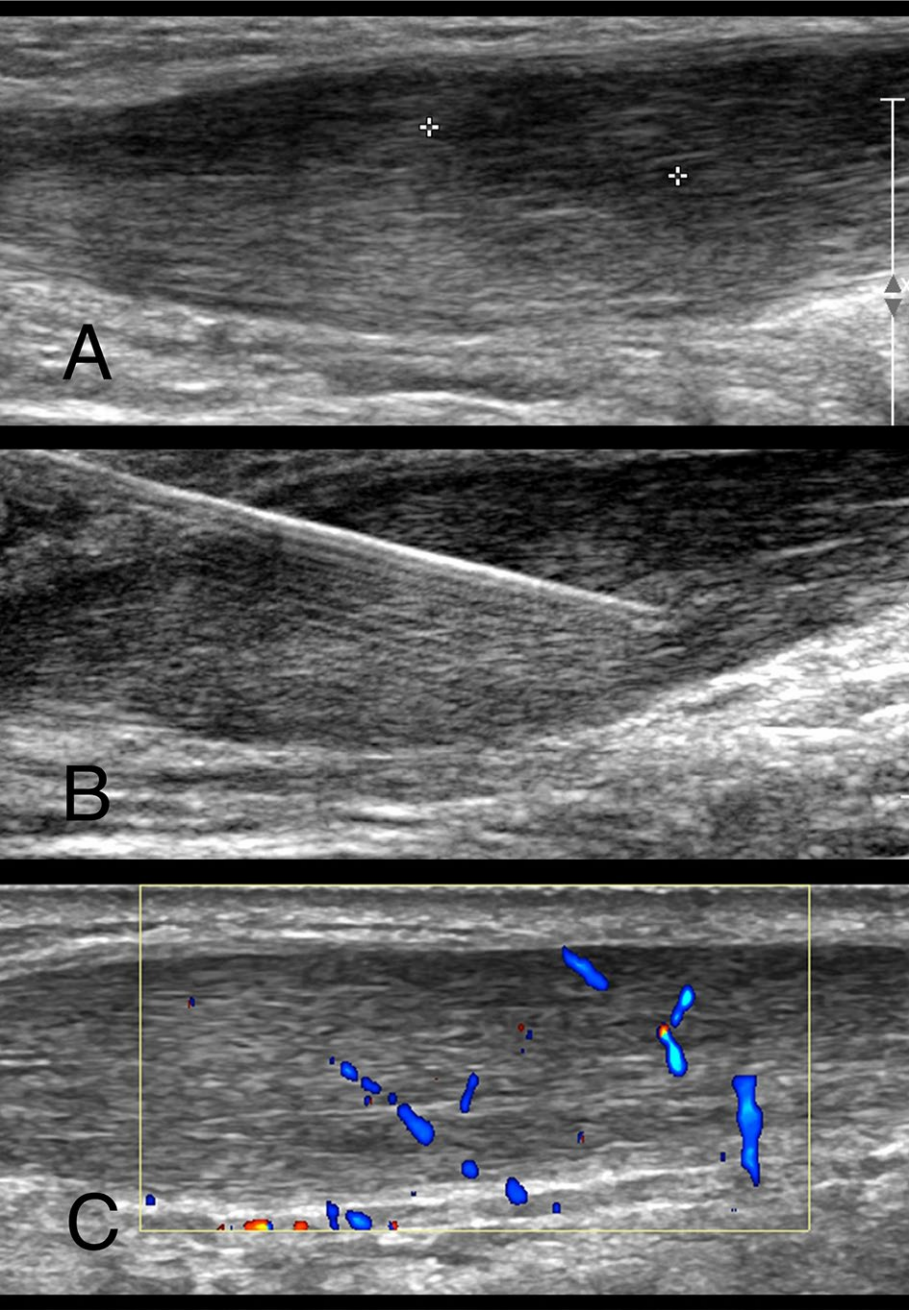
Longitudinal greyscale ultrasound of the right Achilles tendon (**A**) before injection—cursors measuring focal low reflectivity tendinosis); (**B**) similar image showing needle positioning; and (**C**) longitudinal colour doppler mode on grey scale ultrasound six months post injection showing improvement in tendon swelling and improved reflectivity of the tendon.

The primary outcome for this study was the rate of serious adverse reactions. Secondary outcomes included efficacy as determined by the VISA-A, VAS Pain, MOXFQ, EQ-5D-5L, EQ-VAS, and SAS, and overall “success at 24 weeks,” defined as all of these: (i) reduction of ≥ 20 points in VAS pain^43^; (ii) improvement in SAS score; and (iii) increase of > 12 points in VISA-A score (i.e., the minimal clinically important difference, MCID)^44^. Ultrasound outcomes included maximum anteroposterior tendon thickness, lesion distance from the calcaneum, level of neovascularisation, and UTC fibre type (Fig. 4).

### Statistical analysis

Continuous variables are reported as means and standard deviations if normally distributed and as medians and interquartile (IQ) ranges if not normally distributed. Categorical variables are reported as frequencies and percentages. The primary safety endpoint and the secondary endpoint of overall success were calculated with exact binomial confidence intervals.

Exploratory analyses were performed to examine changes in secondary outcomes from baseline to postoperative time points, using a paired t-test (normal distribution) or Wilcoxon matched-pairs test (not normally distributed). All variables were continuous.

Inter-rater reliability of conventional ultrasound measurements was evaluated using the intra-class correlation (ICC) for continuous measurements and the weighted kappa statistic for ordinal measurements. Data from all four time points were included in each analysis.

Associations between UTC changes (from baseline to each subsequent time point) and continuous ultrasound measurements were analyzed using Spearman’s Rank correlation. A p-value < 0.05 was considered statistically significant.

### Ethical approval statement

This study was approved by the National Research Ethics Service Committee (London, Harrow; reference 13/LO/1670) and was registered at clinicaltrials.gov (NCT02064062) on the 17/02/2014.

## Data Availability

Raw data were generated at Royal National Orthopaedic Hospital NHS Trust. Derived data supporting the findings of this study are available from the corresponding author AG on request.
